# EARLY-LIFE ADVERSITY AND ADULT MENTAL AND BEHAVIORAL HEALTH OUTCOMES: AN INTERSECTIONAL APPROACH

**DOI:** 10.1093/geroni/igz038.2560

**Published:** 2019-11-08

**Authors:** Chioun Lee, Lexi Harari, Jennifer Coons

**Affiliations:** 1 University of California-Riverside, Riverside, California, United States; 2 California State University, Fullerton, California, United States

## Abstract

There is much research confirming the link between experiencing early life adversities (ELAs) and adverse health outcomes in adulthood. Further, experiencing co-occurring ELAs rather than one ELA appears to be the rule rather than the exception, and leads to poorer health outcomes. However, information on how ELAs cluster and differentially harm the health of different intersectional groups is lacking. The stress process model suggests that intersectional configurations of race and gender are differentially exposed to ELAs and as a result, some groups suffer worse outcomes than others. We examine the risks of experiencing different clusters of ELAs—low childhood socioeconomic status (SES), family instability, and child abuse—among four intersectional groups (white men, white women, Black men, Black women). We also investigate whether experiencing these ELAs is responsible for the association between having a particular racial and gender configuration and adverse mental and behavioral health outcomes. Data come from a subsample of the Midlife in the U.S. Study (n = 2,076). Black men and women have the highest risk of experiencing all three ELA configurations. Men, regardless of race, have a higher risk of experiencing low SES while women are more vulnerable to low SES/family instability and all three ELAs. Black men and women appear to suffer the worst mental health outcomes, while Black and white men experience more drug/alcohol abuse than their female counterparts. ELAs, especially the co-occurrence of all three ELAs, partially mediate most of these associations, but more so for women than men.

